# Pre-hospital management of acute stroke patients eligible for thrombolysis – an evaluation of ambulance on-scene time

**DOI:** 10.1186/s13049-018-0580-4

**Published:** 2019-01-09

**Authors:** Nicolas Drenck, Søren Viereck, Josefine Stokholm Bækgaard, Karl Bang Christensen, Freddy Lippert, Fredrik Folke

**Affiliations:** 1 Emergency Medical Services Copenhagen, University of Copenhagen, Copenhagen, Denmark; 2 Department of Biostatistics, University of Copenhagen, Copenhagen, Denmark; 3 Department of Cardiology, Gentofte University Hospital, Copenhagen, Denmark

**Keywords:** Emergency medical services, Stroke, Pre-hospital stroke management, Stroke on-scene time, Pre-hospital delay, Thrombolysis, Cerebrovascular disease, Ischemic stroke

## Abstract

**Background:**

Stroke is a leading cause of death and disability with effective treatment, including thrombolysis or thrombectomy, being time-critical for favourable outcomes. While door-to-needle time in hospital has been optimized for many years, little is known about the ambulance on-scene time (OST). OST has been reported to account for 44% of total alarm-to-door time, thereby being a major time component. We aimed to analyse ambulance OST in stroke patients eligible for thrombolysis and identify potential areas of time optimization.

**Methods:**

A study-specific registration form was developed to record detailed information about OST consumption in cases where the Emergency Medical Services (EMS) suspected a stroke from July 2014–May 2015. Registration forms were completed by ambulance personnel and included details on estimated time spent: 1) localising patient, 2) clinical examination, 3) consulting with the on-call neurologist, 4) mobilising patient to the ambulance, 5) treatment in ambulance before departure. Additionally, estimated total OST was noted. For patients found eligible for further evaluation at a stroke centre, time points were analysed using multivariate Poisson regressions.

**Results:**

A total of 520 cases were included. The median OST was 21 min (Interquartile Range (IQR) 16–27). Time consumption was significantly lower (17 vs 21 min, *p* = 0.0015) when electrocardiography (ECG) was obtained in-hospital instead of on-scene, when intravenous (IV) access was established during transportation instead of before transportation (17 vs 21 min, *p* < 0.0001), and when the quality of communication with the stroke centres was rated as “good” as opposed to “acceptable/poor” (21 vs 23 min, *p* = 0.014). Neither the presence of relatives nor ambulance trainees had a significant effect on OST.

**Conclusions:**

In-hospital ECG recording and IV cannulation during transport were found to reduce OST, while “acceptable/poor” communication was found to prolong OST relative to “good” communication. These components of pre-hospital stroke management represent potential opportunities for lowering OST with relatively simple changes, which could ultimately lead to earlier treatment and better patient outcome.

**Trial registration:**

Unique identifier: NCT02191514.

**Electronic supplementary material:**

The online version of this article (10.1186/s13049-018-0580-4) contains supplementary material, which is available to authorized users.

## Background

In 2016, stroke accounted for 5.78 million deaths globally, making it the second leading cause of death [1]. In the United States, a stroke occurs every 40 s and every 4 min someone dies of stroke, totalling around 140,000 stroke related deaths annually. Many survivors will have permanent disabilities, and it is estimated that stroke associated costs amount to $34 billion annually in health care, medication, and lost productivity in the United States [2]. Each year 12,000 people suffer from a stroke in Denmark, and only 85% survive past the first month [3].

Strokes are divided into two types; ischemic strokes, comprising 85%, and haemorrhagic strokes, comprising, 15%. In all cases of stroke, rapid diagnostic work-up is key to verifying the final diagnosis and ensuring that the appropriate treatment is instituted as quickly as possible. For haemorrhagic strokes direct treatment of the underlying cause is often not readily available in contrast to ischemic strokes. It is well-known that time is of the essence in ischemic stroke as the ischemic insult can progress to rapid and irreversible brain damage [4]. Treatment of ischemic stroke aims to preserve tissue in the ischemic penumbra, by restoring blood flow to the affected areas. This is often accomplished through the administration of intravenous (IV) recombinant tissue-type plasminogen activator (rtPA), which must be initiated as early as possible, but within 4.5 h of symptom onset in order to maximize its effect while minimizing the risk of haemorrhage [5]. This is 4.5 h time frame is followed in recommendations in both American and Danish guidelines [3, 6].

When the ambulance services arrive on-scene and suspect a stroke, standard procedure includes recording of a 12-lead electrocardiograph (ECG), establishment of two IV accesses, measurement of vital signs, and evaluating the patient for pre-defined symptoms of stroke. In a previous study based on data from the Capital Region of Denmark, on-scene time (OST) has been reported to account for 44% of the total alarm-to-door time (median of 18 min) [7].

Traditionally, much effort was devoted to optimizing the in-hospital treatment and management of strokes, reducing the time from hospital arrival to treatment. However, over the past decade research on pre-hospital stroke management has increased steadily, but little is known about time spent on-scene in the pre-hospital setting for stroke patients eligible for thrombolysis. Therefore, the aim of this study was to analyse the time spent on-scene by the ambulance personnel when stroke was suspected, and the stroke centre neurologist found the patient eligible for further examination at the stroke centre. We hypothesized that performing procedures such as ECG and IV cannulation during transport or in-hospital would be linked to lower OSTs. Likewise, we hypothesized that simultaneously consultation with the stroke centre and carrying out procedures as well as having more available personnel present would be associated with lower OSTs. Through our analysis we hoped to identify areas in the on-scene stroke workflow where optimization could result in lower OSTs and thereby a shortened overall time from symptom onset to treatment.

## Methods

We carried out a cross-sectional study based on prospectively collected data from registration forms completed by EMS personnel on-scene whilst caring for patients with suspected stroke who were potential candidates for thrombolysis.

The setting of the study was the Capital Region of Denmark, which has a population of 1.83 million inhabitants. It includes the city of Copenhagen and surrounding suburbs and covers an area of 2559 km^2^ [8]. When a stroke occurs, the patient or his/her relatives must first recognise and act upon the symptoms by alerting the EMS by phone. Second, the EMS dispatch centre sends a -priority “A” ambulance response (highest level of urgency) and once on scene, ambulance personnel evaluate the patient for symptoms and signs of stroke. The ambulance personnel must then confer with the on-call neurologist at one of the region’s two stroke centers to assess whether the patient is eligible for an emergency neurological examination and neuroimaging in order to further evaluate eligibility for thrombolysis. In addition to a detailed medical history, standard operating protocols as well as national guidelines mandate that the ambulance personnel should measure vital signs and blood glucose, obtain a 12-lead ECG, establish two IV accesses and administer supplementary oxygen [9]. Once at the hospital, neuroimaging is performed and a choice of treatment strategy is made.

The study is part the research project “Prehospital management of stroke patients by the EMS” which is protocolled online at www.clinicaltrials.gov (NCT02191514). Approval from the Danish Data Protection Agency (2007-58-0015) was obtained. Approval from the regional ethics committee was not required (H-4-2014-FSP). The ethics committee deemed that informed consent was not required as the data collected concerned personnel workflow and no health related or personally identifiable information was gathered.

### Participants

All patients above the age of 18 treated by ambulance personnel in the Capital Region of Denmark from July 1st, 2014 to May 1st, 2015 who were suspected of having a stroke and who were potential candidates for thrombolysis were included. The 4.5-h time limit for thrombolysis had to be within reach and stroke suspicion had to be raised by ambulance personnel by finding stroke symptoms including unilateral paresis/paralysis and/or sensory deficits, speech impairment, or hemianopsia. Further, the on-call neurologist at the stroke centre decided whether further diagnostic examination at the stroke centre was warranted.

Patients who were ultimately not considered eligible for further evaluation at the stroke centre after conferring with the on-call neurologist were subsequently excluded by the authors. This was done as ambulance personnel may thus have considered the situation less urgent, as the 4.5 h time-frame had then become irrelevant, perhaps leading to longer OSTs. However, no information on reasons for non-eligibility was recorded.

### Data collection

**Fig. 1:**
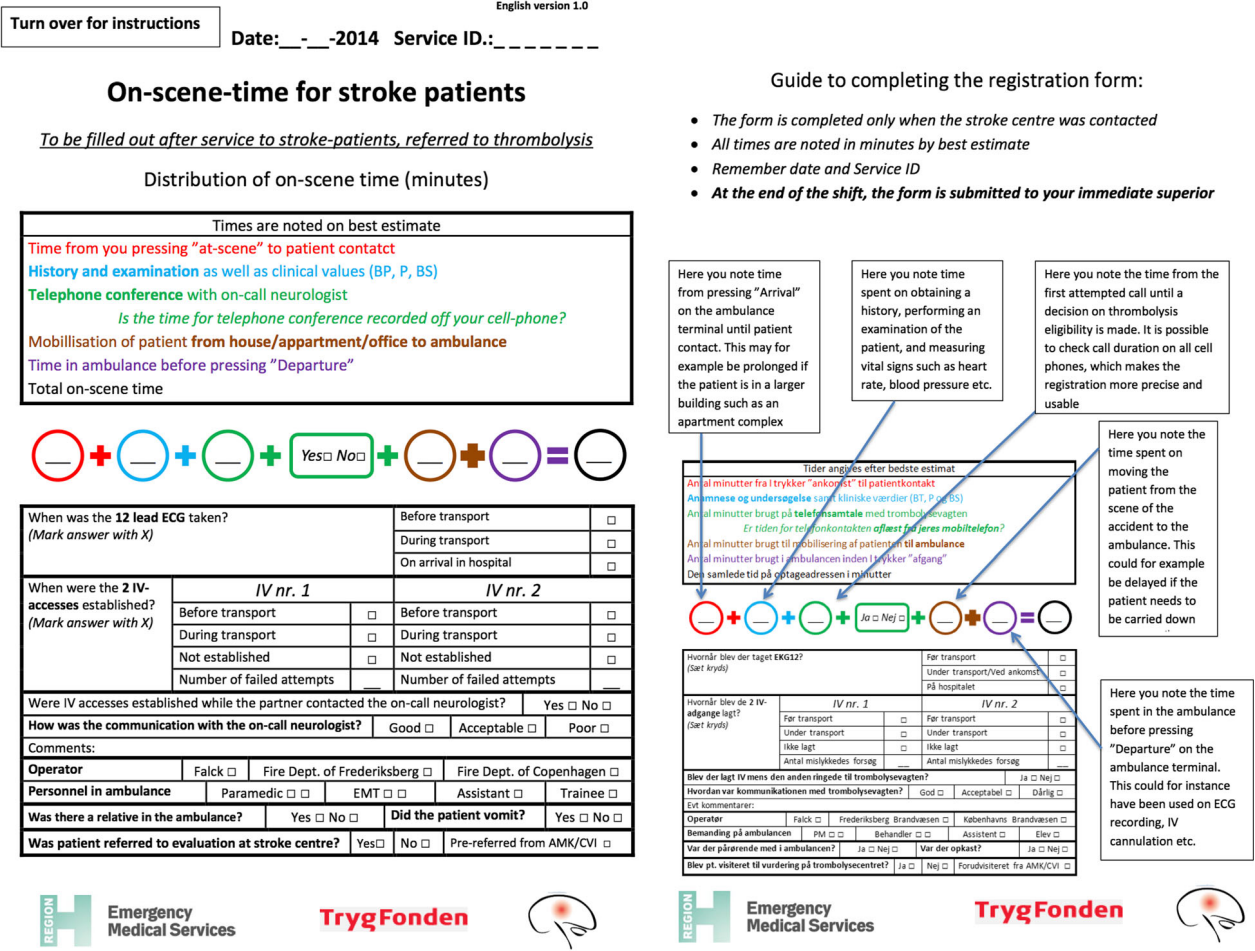
Page 1 and page 2 of the registration form used for data collection

A study-specific registration form was developed (Fig. 1). Ambulance personnel completed the form whenever they suspected a stroke eligible for thrombolysis and contacted the on-call neurologist (regardless of the dispatch code received from EMS dispatchers). Time consumption was registered for five different points (termed “T1” to “T5”, respectively) of the on-scene time: 1) time spent localising the patient (termed “T1”), 2) time spent on obtaining a medical history and measuring vital signs as well as assessing the patient for neurological deficits and stroke symptoms (termed “T2”), 3) time spent conferring with the on-call neurologist (termed “T3”), 4) time spent mobilising the patient to the ambulance (termed “T4”), 5) time spent in the ambulance before departure (termed “T5”). Furthermore, total OST spent was registered (“total OST”, the sum of time T1-T5).

Circumstantial data included timing of ECG and IV cannulation, whether the on-call neurologist was contacted during cannulation or not, the quality of the communication with the on-call neurologist (good/acceptable/poor), the ambulance provider dispatch to the case (Fire Department of Copenhagen/Frederiksberg or Falck), the composition of the crew manning the ambulance (2–3 man crew composed of a combination of (in descending order of training level): paramedics, advanced ambulance assistants, ambulance assistants, and trainees), presence of a relative in the ambulance, and whether the patient was considered eligible for thrombolysis and was taken to the stroke centre for further evaluation. Vomiting has been reported to occur in 14.5% of all strokes and was therefore included as circumstantial data to allow for adjustment of the statistical analysis for the effect that vomiting might have on time consumption [10].

Patients with incomplete registration forms on any of the five time points, T1-T5, were subsequently excluded. Forms with missing registration of total OST were not excluded if T1-T5 had been completed. Forms with missing circumstantial data were not excluded (see Additional file 1: Table S1).

### Statistics

The five parts of the OST (T1-T5) as well as the total OST, calculated as sum of all five time registrations (T1-T5), were considered as outcome variables. The circumstantial data recorded were considered as explanatory variables and used to explain variations in outcome variables. For the explanatory variable “Operator”, two of the groups, Fire Department of Copenhagen and Fire Department of Frederiksberg (both public operators) were combined into one group in order to focus on the differences between public operators and the major private ambulance operator, Falck. Similarly, for the variable “Quality of communication” the groups “acceptable” and “poor” were combined into a single group, “acceptable/poor”, due to a low number of observations in the “poor” group.

The impact of the explanatory variables on the outcome variable was analysed using multivariate Poisson regression with an added scale parameter to account for over-dispersion [11]. Results are presented as median times, interquartile-ranges (IQR), and rate ratios (RR) with corresponding 95% confidence intervals (CI).

Data analysis was carried out using the SAS 9.4 (2014) software package.

## Results

**Fig. 2:**
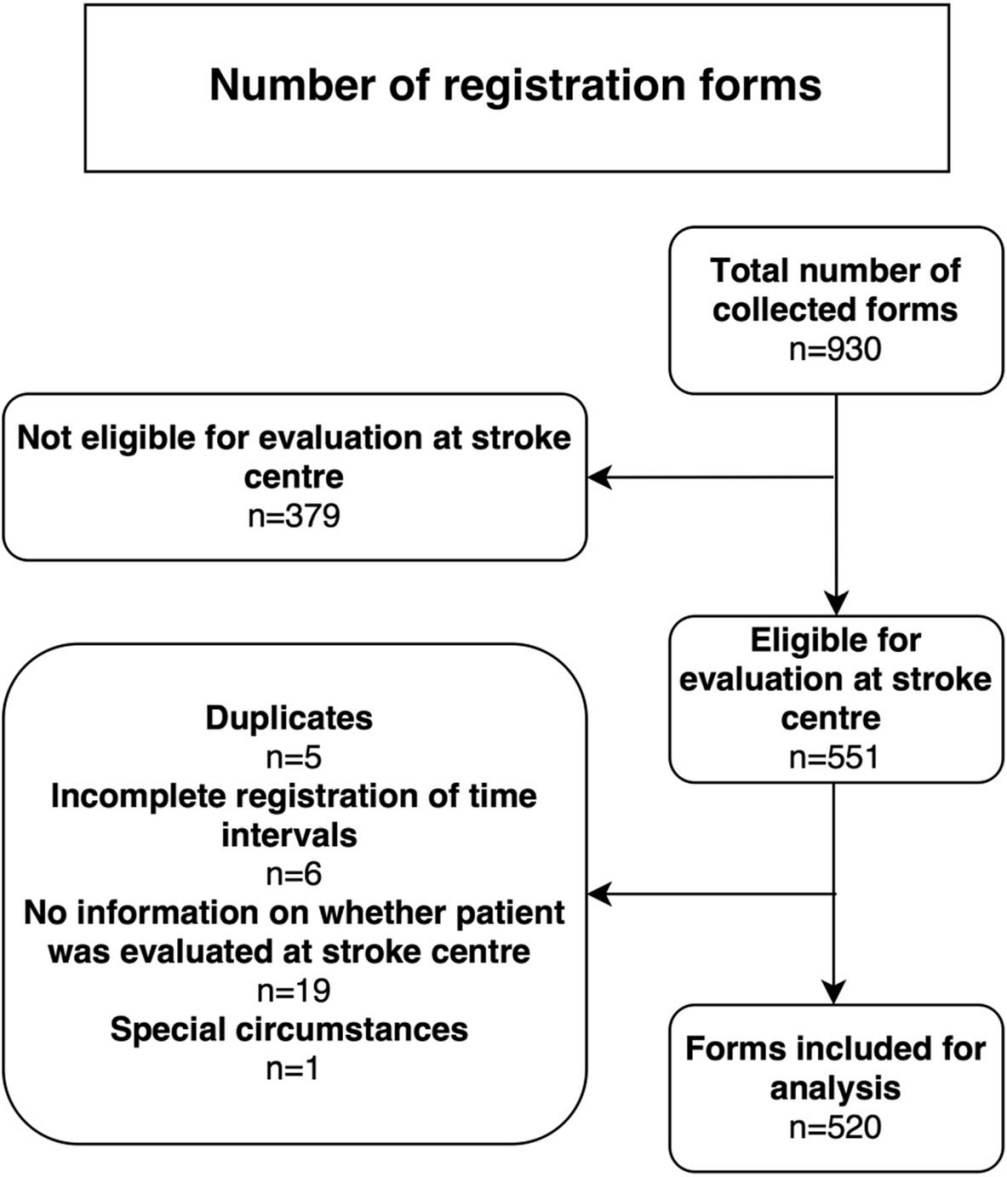
Flowchart depicting the reasons for exclusion of registration forms. Patients considered not eligible for evaluation at the stroke center were excluded. However, no reasons for non-eligibility were recorded

A total of 930 registration forms were collected. After the removal of duplicates, incomplete forms, and patients who were not considered eligible for further evaluation at a stroke centre, 520 registration forms were included for analysis (Fig. 2).

The median total OST was 21 min (IQR: 16–27). This included a median “T1” of 2 min (IQR: 1–2), a median “T2” of 5 min (IQR: 4–9), a median “T3” of 3 min (IQR: 2–5), a median “T4” of 3 min (IQR 2–5), and a median “T5” of 5 min (IQR: 2–8). The distributions of T1- T5 and total OST were skewed (non-normal).

### Total on-scene time

We found in-hospital ECG to significantly reduce total OST compared with ECG obtained on-scene (median 17 vs. 21 min, estimated time reduction was 24%, RR = 0.76, 95% CI: 0.64 to 0.90, *p* = 0.0015). No significant reduction in OST was shown when ECGs were obtained during transport relative to on-scene (RR = 1.00, 95% CI: 0.92 to 1.08, *p* = 0.92). Establishing the first IV access during transport was found to reduce OST by 19% compared with cases where access was established on-scene (median 17 vs. 21 min, RR = 0.81, 95% CI: 0.73 to 0.89, *p* < 0.0001). Communication rated as “acceptable/poor” was found to be associated with a 10% increase in total OST relative to those rated as “good” (RR = 1.10, 95% CI: 1.02 to 1.18, *p* = 0.014), with median times of 23 and 21 min, respectively. Additionally, patient vomiting was found to be a significant predictor of a longer total OST (25 vs. 21 min, RR = 1.16, 95% CI: 1.04 to 1.29, *p* = 0.0099) (Table 1).

**Table 1:** Effects of explanatory variables on total on-scene time

Variable	Group	*N*	Median time (IQR)	Rate ratio (95% CI)	*p*-value
ECG	Before transport	326	21 (17–27)	1.00 (−)	–
	During transport	86	21 (14–28)	1.00 (0.92–1.08)	0.92
	At hospital	20	17 (15–20.5)	0.76 (0.64–0.90)	0.0015
First IV access	Before transport	385	21 (17–27)	1.00 (−)	–
	During transport	69	17 (13–23)	0.81 (0.73–0.89)	<.0001
	Not established	13	28 (23–29)	1.09 (0.91–1.30)	0.36
IV access during conference with stroke centre	Yes	174	20 (15–28)	1.00 (−)	–
	No	258	21.5 (16–27)	1.05 (0.98–1.13)	0.17
Quality of communication	Good	337	21 (16–26)	1.00 (−)	–
	Acceptable/Poor	95	23 (17–29)	1.10 (1.02–1.18)	0.014
Operator	Fire Departments^a^	67	21 (16–28)	1.05 (0.96–1.15)	0.31
	Falck	365	21 (16–27)	1.00 (−)	–
Presence of paramedic	Yes	190	21 (16–28)	0.99 (0.93–1.05)	0.69
	No	242	21 (16–27)	1.00 (−)	–
Presence of relative	Yes	159	21 (16–27)	0.98 (0.92–1.05)	0.65
	No	273	21 (16–27)	1.00 (−)	–
Vomit	Yes	35	25 (20–29)	1.16 (1.04–1.29)	0.0099
	No	397	21 (16–27)	1.00 (−)	–
Presence of trainee	Yes	38	20 (16–25)	0.92 (0.82–1.04)	0.18
	No	394	21 (16–27)	1.00 (−)	–

The analyses were adjusted for all variables listed above as well as the timing of second IV access. The analysis of the first IV access was not adjusted for timing of the second IV access. Second IV access is not listed, as no model to isolate the effect of the second IV access could be developed

The number of observations in each group includes only those included in the multivariate analysis (i.e. only those with complete data in all the variables used in the analysis). Thus, all 520 observations were not necessarily used

^a^Fire Departments: Fire Department of Copenhagen and Fire Department of Frederiksberg

The presence of a relative in the ambulance did not affect the total OST (RR = 0.98, 95% CI: 0.92 to 1.05, *p* = 0.65). Similarly, comparison of cases with and without an ambulance trainee present showed no significant difference (RR = 0.92, 95% CI: 0.82 to 1.04, *p* = 0.18). The presence of a paramedic did not affect OST either (RR = 0.99, 95% CI: 0.93 to 1.05, *p* = 0.69) (Table 1). For detailed results and group sizes, please refer to Table 1.

### Individual time points (T1-T5)

When analysing the individual time intervals (T1-T5), we found no significant differences in T1 (localising the patient) between any groups (see Additional file 1: Table S2). For T2 (medical history, measurement of vital signs and examination of patient), presence of a paramedic and the operators, Fire Department of Copenhagen and Fire Department of Frederiksberg, were associated with higher time consumption, while cannulation during conference with the stroke centre was linked to reduced time consumption (see Additional file 1: Table S3). T3 (conference with on-call neurologist) was found to be significantly longer with the operators, Fire Department of Copenhagen and Fire Department of Frederiksberg, and communication between stroke centre and EMS rated as either “acceptable/poor” was associated with a considerably higher time consumption than “good” communication. IV cannulation during conference with the stroke centre was, however, not found to significantly affect T3 (see Additional file 1: Table S4). T4 (mobilising patient to ambulance) was found to be significantly prolonged by the patient vomiting (see Additional file 1: Table S5). T5 (time in ambulance before departure) was reduced by in-hospital ECGs, establishment of the first IV access during transport, presence of a paramedic, and cannulation during conference with the stroke centre (see Additional file 1: Table S6).

## Discussion

In this study we found that the median on scene time was 21 min (IQR: 16–27). In-hospital ECG recording was associated with significantly lower OSTs than on-scene recording. Similarly, establishing the first IV access during transport was linked to lower OSTs than doing so on-scene. However, both ECG during transport and IV cannulation in-hospital was found to yield no different OSTs than their respective on-scene counterparts. We also found that both communication rated as “acceptable/poor” and patient vomiting were linked to significantly longer OSTs than “good” communication and no patient vomiting, respectively. Additionally, neither relatives nor ambulance trainees present on-scene or in the ambulance were found to affect OST when compared to no relatives or ambulance trainees, respectively.

Currently, Danish stroke guidelines do not specify a benchmark OST for responding to stroke [9]. Instead, general recommendations to examine and treat the patient as fast as possible are issued. However, in contrast to these recommendations, an American study reported that EMS systems that specified a target on-scene time limit had lower OSTs than EMS systems with only general or no recommendations regarding OST [12].

As for the relevance of striving to reduce on-scene time consumption by even just a few minutes, it has been reported that a focused effort resulting in a 10 min reduction of median time from in-hospital arrival to thrombolysis initiation (77 min before vs. 67 min after) was associated with significant decreases in in-hospital all-cause mortality and symptomatic intracranial haemorrhage within 36 h as well as an increase in the percentage of patients being discharged to home [13]. By extrapolation these findings suggest that even modest reductions in OST may improve the prognosis for patients according to the notion that faster thrombolysis yields better outcomes. In this context it is also worth noting that a substantial part of the time from symptom onset to treatment is, in many cases, the time that passes from symptom onset until altering of the EMS [14–16]. Educational stroke awareness therefore remains fundamental in the effort to bring down the time from symptoms to treatment, giving patients the best chance of a favourable prognosis.

With ongoing efforts to reduce delay from symptom onset to treatment it is also important to keep in mind the level of overtriage of stroke-like presentations. Overtriage of these “stroke-mimics” can be hypothesized to increase in frequency with growing emphasis on limiting time consumption and use of rough stroke screening tools which have been reported to have wide specificity ranges [17]. Studies in overtriage of stroke-mimics are therefore highly necessary to ensure that pre- and in-hospital resources are utilized optimally.

Our finding of a median OST of 21 min is comparable to earlier Finnish and Danish studies, which report OSTs between 18 and 25 min, [7, 18] while an American study has reported OSTs as low as 15 min [12]. Various studies of Mobile Stroke Units (MSUs) have also reported on on-scene time consumption [19–21]. The OSTs reported in these studies have generally been longer than our findings, but direct comparisons should be avoided, as the MSU facilitates on-scene imaging diagnostics and thrombolytic treatment, which may prolong OST. Additionally, the concept of OST is fundamentally different in these studies, as the on-scene phase becomes an integral part of the treatment phase.

Not surprisingly, obtaining an ECG in-hospital instead of on-scene reduces the total OST (24% reduction). This does not necessarily translate to a reduction in overall alarm-to-treatment time, as the time spent obtaining an ECG is instead added to the in-hospital time. It could, however, be hypothesized that ECG recording in the stroke centre may be quicker due to a more organized setting with more personnel accessible. Ultimately, it is important to note that for the reduction in OST demonstrated in this study to benefit the patient, it needs to be associated with reductions in the total time from symptom onset to hospital treatment. Studies that encompass all phases of stroke management from onset to treatment are therefore highly relevant and needed. It has been reported that ECG recording prior to neuroimaging was linked to a 6 min increase in time from arrival in-hospital to neuroimaging and that patients were 3.67 times more likely to receive rtPA treatment within 60 min of arriving in-hospital when ECG recording was performed after neuroimaging [22]. A systematic review of the use of 12-lead ECGs in pre-hospital stroke care failed to identify any evidence to back up the recommendation that ECG be recorded on-scene as no studies in the pre-hospital setting were found. The study concludes that investigation of the potential diagnostic benefits or harms from treatment delays of pre-hospital ECG is needed before recommendations whether or not to record a pre-hospital ECG can be made [23]. Conversely, in a recent prospective study of 186 stroke patients without a prior history of atrial fibrillation, almost 10% showed signs of atrial fibrillation on the pre-hospital ECGs. However, in 11% of these the atrial fibrillation was no longer present in-hospital. The authors therefore argue, that pre-hospital ECG recording is important, despite of the extra time spent on-scene [24].

Establishing the first IV access during transport was shown to reduce OST by almost 20% and demonstrates and important opportunity of time reduction. It could be argued that cannulation during transport is more difficult and may also pose a risk to both patient and ambulance personnel [18]. Nonetheless, balancing potential risks and rewards, it should at least be considered to establish IV accesses during transport if possible in order to reduce OST and alarm-to-treatment time.

This study also demonstrated that communication between EMS and stroke centre rated “acceptable/poor” compared with “good” was linked to a 2-min increase in median OST. It is also noteworthy that when looking at “T3” alone (duration of conference with stroke centre), we find that “acceptable/poor” communication compared with “good” communication was significantly associated with a 45% increase in “T3”. These findings call for attention and further research into communication between EMS and stroke centres.

A recent Finnish study aiming to reduce OST employed a training programme to enhance ambulance personnel performance [18]. The training programme specified that emergency medical technicians and paramedic students should not examine nor practice procedures on suspected thrombolysis candidates. Our results show that the presence of ambulance trainees did not prolong OST. It does, however, remain unclear to what extent the trainees were actively performing procedures and helping and clear conclusions about the role of trainees cannot be drawn. Similarly, the presence of relatives was found not to affect total OST or T1-T5.

Not surprisingly, we found that if a patient vomited it was linked to a significant increase in total OST. Increased awareness of vomiting as an important predictive factor for prolonged OSTs may be important to ensure swift handling of the situation when it does happen and thus minimise the increase in OST.

### Strengths and limitations

The method of data collection utilized by this study enables us to map out time consumption in a more detailed way than could be achieved through existing electronic records of ambulance arrival and departure. To our knowledge, no previous study of pre-hospital stroke management has employed similarly detailed methods to investigate OST and as a result this study provides a unique insight into the dynamics of the on-scene stroke management. Additionally, the large number of participants included in the study strengthens the analyses that have been carried out as it ensures representative sampling of data in the setting of the study. Our study relied on ambulance personnel to complete the registration forms on best estimate while caring for stroke patients in a possibly hectic environment. This may have influenced the time perception of the ambulance personnel and thus the accuracy of the registrations. However, as the same method of data collection was used for all data and due to the large data sample size, this uncertainty should be of limited significance. Exclusion of forms with one or more missing time registrations carries a risk of bias, as for example extraordinarily long or short time intervals may have been excluded; long intervals may have been consciously underreported for fear of “bad” results and the registration of very short intervals may have been forgotten if the situation was dealt with smoothly and quickly.

Our study would have been further strengthened, if we had been able to analyse whether the reductions in OST found corresponded to reductions in onset-to-treatment times and to what degree the level of stroke-mimic overtriage was influenced by OST duration.

## Conclusions

In this study we found that OST was reduced by in-hospital ECG recording and IV cannulation during transport to the hospital. Communication rated as “acceptable/poor” was associated to longer OSTs than “good” communication, and vomiting was found to significantly increase OST. The presence of relatives and trainees was found to have no effect on OST.

These components of the stroke response represent potential areas of optimization and reduction of OST which could lead to earlier treatment and better outcome for patients.

## Additional file

Additional file 1: **Table S1.** contains information regarding missing circumstantial data in the registration forms collected. **Tables S2-S6.** contains additional results of analyses of the explanatory variables’ effects on specific parts of total on-scene time (T1-T5). (DOCX 35 kb)

## Abbreviations

CI: Confidence interval
ECG: Electrocardiography
EMS: Emergency medical services
IQR: Interquartile range
IV: Intravenous
OST: On-scene time
RR: Rate ratio
rtPA: Recombinant tissue-type plasminogen activator
