# Supplementary material for: Pre-hospital management of acute stroke patients eligible for thrombolysis – an evaluation of ambulance on-scene time
Source: Scand J Trauma Resusc Emerg Med. 2019 Jan 9;27:3. doi: 10.1186/s13049-018-0580-4 (PMC6327613; doi:10.1186/s13049-018-0580-4)
Supplement: Supplementary file 1 — Table S1. contains information regarding missing circumstantial data in the registration forms collected. Tables S2-S6. contains additional results of analyses of the explanatory variables’ effects on specific parts of total on-scene time (T1-T5). (DOCX 35 kb) [file 13049_2018_580_MOESM1_ESM.docx]

Additional file 1

**Supplemental methods:** none

**Supplemental tables:** 6

**Supplemental figures:** none

**Supplemental video:** none

## Additional file 1

**Table S1** Number of included registration forms with missing data in circumstantial data variables.

| **Circumstantial data variable** | **Number missing** |
| --- | --- |
| ECG | 9 |
| First IV access | 15 |
| Second IV access | 49 |
| IV access was established during conference with stroke centre | 13 |
| Quality of communication with stroke centre | 10 |
| Operator | 1 |
| Presence of paramedic | 0 |
| Presence of relatives | 1 |
| Vomit | 9 |
| Presence of trainee | 0 |

**Table S2** Effects of variables on time spent localising the patient (Time 1)

| **Variable** | **Group** | **N** | **Median time (IQR)** | **Rate ratio (95% CI)** | **p-value** |
| --- | --- | --- | --- | --- | --- |
| Operator | Fire Departments* | 72 | 2 (1-2) | 0.98 (0.82-1.19) | 0.87 |
|  | Falck | 447 | 2 (1-2) | 1.00 (-) | - |
| Presence of paramedic | Yes | 215 | 2 (1-3) | 1.06 (0.93-1.21) | 0.38 |
|  | No | 304 | 1 (1-2) | 1.00 (-) | - |
| Presence of trainee | Yes | 43 | 1 (1-2) | 0.87 (0.68-1.12) | 0.28 |
|  | No | 476 | 2 (1-2) | 1.00 (-) | - |

**Table S2 legend and footnotes**

The analyses were adjusted for all variables listed above.

The number of observations in each group includes only those included in the multivariate analysis (i.e. only those with complete data in all the variables used in the analysis). Thus, all 520 observations were not necessarily used.

**** Fire Departments:*** *Fire Department of Copenhagen and Fire Department of Frederiksberg*

**Table S3** Effects of variables on time spent on history and examination of patient (Time 2)

| **Variable** | **Group** | **N** | **Median time (IQR)** | **Rate ratio (95% CI)** | **p-value** |
| --- | --- | --- | --- | --- | --- |
| ECG | Before transport | 329 | 5 (4-9) | 1.00 (-) | - |
|  | During transport | 89 | 5 (3-8) | 0.97 (0.84-1.11) | 0.65 |
|  | At hospital | 20 | 4.5 (3.5-8) | 0.84 (0.64-1.11) | 0.22 |
| First IV access | Before transport | 391 | 5 (4-10) | 1.00 (-) | - |
|  | During transport | 71 | 5 (3-7) | 0.87 (0.74-1.02) | 0.091 |
|  | Not established | 13 | 6 (5-9) | 0.88 (0.63-1.24) | 0.48 |
| IV access during conference with stroke centre | Yes | 177 | 5 (3-8) | 1.00 (-) | - |
|  | No | 261 | 5 (4-9) | 1.14 (1.01-1.28) | 0.033 |
| Operator | Fire Departments* | 68 | 7 (5-10) | 1.35 (1.17-1.56) | <.0001 |
|  | Falck | 370 | 5 (4-8) | 1.00 (-) | - |
| Presence of paramedic | Yes | 191 | 6 (4-10) | 1.13 (1.01-1.26) | 0.029 |
|  | No | 247 | 5 (4-8) | 1.00 (-) | - |
| Presence of relative | Yes | 160 | 5 (4-8) | 0.93 (0.83-1.05) | 0.25 |
|  | No | 278 | 5 (4-9) | 1.00 (-) | - |
| Vomit | Yes | 36 | 6 (4-10) | 1.14 (0.94-1.38) | 0.19 |
|  | No | 402 | 5 (4-9) | 1.00 (-) | - |
| Presence of trainee | Yes | 38 | 5 (4-7) | 0.98 (0.80-1.20) | 0.85 |
|  | No | 400 | 5 (4-9) | 1.00 (-) | - |

**Table S3 legend and footnotes**

The analyses were adjusted for all variables listed above as well as the timing of second IV access. The analysis of the first IV access was not adjusted for timing of the second IV access. Second IV access is not listed, as no model to isolate the effect of the second IV access could be developed.

The number of observations in each group includes only those included in the multivariate analysis (i.e. only those with complete data in all the variables used in the analysis). Thus, all 520 observations were not necessarily used.

**** Fire Departments:*** *Fire Department of Copenhagen and Fire Department of Frederiksberg*

**Table S4** Effects of variables on time spent conferring with stroke centre neurologist (Time 3)

| **Variable** | **Group** | **N** | **Median time (IQR)** | **Rate ratio (95% CI)** | **p-value** |
| --- | --- | --- | --- | --- | --- |
| IV access during conference with stroke centre | Yes | 192 | 3 (3-5) | 1.00 (-) | - |
|  | No | 305 | 3 (2-5) | 0.94 (0.85-1.04) | 0.21 |
| Quality of communication | Good | 397 | 3 (2-4) | 1.00 (-) | - |
|  | Acceptable/Poor | 100 | 5 (3-6) | 1.45 (1.29-1.62) | <.0001 |
| Operator | Fire Departments* | 70 | 4 (3-5) | 1.21 (1.06-1.38) | 0.0047 |
|  | Falck | 427 | 3 (2-5) | 1.00 (-) | - |
| Presence of paramedic | Yes | 211 | 3 (2-5) | 1.04 (0.94-1.15) | 0.48 |
|  | No | 286 | 3 (2-5) | 1.00 (-) | - |
| Presence of trainee | Yes | 42 | 3 (2-4) | 0.94 (0.78-1.13) | 0.51 |
|  | No | 455 | 3 (2-5) | 1.00 (-) | - |

**Table S4 legend and footnotes**

The analyses were adjusted for all variables listed above.

The number of observations in each group includes only those included in the multivariate analysis (i.e. only those with complete data in all the variables used in the analysis). Thus, all 520 observations were not necessarily used.

**** Fire Departments:*** *Fire Department of Copenhagen and Fire Department of Frederiksberg*

**Table S5** Effects of variables on time spent mobilising patient to ambulance (Time 4)

| **Variable** | **Group** | **N** | **Median time (IQR)** | **Rate ratio (95% CI)** | **p-value** |
| --- | --- | --- | --- | --- | --- |
| Operator | Fire Departments* | 72 | 2 (1-5) | 0.84 (0.67-1.06) | 0.14 |
|  | Falck | 438 | 3 (2-5) | 1.00 (-) | - |
| Presence of paramedic | Yes | 212 | 3 (2-5) | 1.07 (0.92-1.24) | 0.39 |
|  | No | 298 | 3 (2-6) | 1.00 (-) | - |
| Presence of relative | Yes | 190 | 3 (1-6) | 1.04 (0.90-1.22) | 0.58 |
|  | No | 320 | 3 (2-5) | 1.00 (-) | - |
| Vomit | Yes | 41 | 5 (2-8) | 1.38 (1.09-1.75) | 0.0072 |
|  | No | 469 | 3 (2-5) | 1.00 (-) | - |
| Presence of trainee | Yes | 43 | 3 (2-5) | 0.97 (0.74-1.27) | 0.83 |
|  | No | 467 | 3 (2-5) | 1.00 (-) | - |

**Table S5 legend and footnotes**

The analyses were adjusted for all variables listed above as well as the timing of second IV access. The analysis of the first IV access was not adjusted for timing of the second IV access. Second IV access is not listed, as no model to isolate the effect of the second IV access could be developed.

The number of observations in each group includes only those included in the multivariate analysis (i.e. only those with complete data in all the variables used in the analysis). Thus, all 520 observations were not necessarily used.

**** Fire Departments:*** *Fire Department of Copenhagen and Fire Department of Frederiksberg*

**Table S6** Effects of variables on time spent in ambulance before departure (Time 5)

| **Variable** | **Group** | **N** | **Median time (IQR)** | **Rate ratio (95% CI)** | **p-value** |
| --- | --- | --- | --- | --- | --- |
| ECG | Before transport | 326 | 5 (3-8) | 1.00 (-) | - |
|  | During transport | 86 | 4 (2-7) | 0.92 (0.76-1.12) | 0.43 |
|  | At hospital | 20 | 2 (1-4) | 0.47 (0.28-0.77) | 0.0028 |
| First IV access | Before transport | 385 | 5 (3-8) | 1.00 (-) | - |
|  | During transport | 69 | 3 (1-5) | 0.55 (0.43-0.71) | <.0001 |
|  | Not established | 13 | 8 (4-12) | 1.26 (0.87-1.80) | 0.22 |
| IV access during conference with stroke centre | Yes | 174 | 4 (2-7) | 1.00 (-) | - |
|  | No | 258 | 5 (3-8) | 1.18 (1.00-1.38) | 0.047 |
| Quality of communication | Good | 337 | 5 (2-8) | 1.00 (-) | - |
|  | Acceptable/Poor | 95 | 5 (2-9) | 1.14 (0.96-1.35) | 0.15 |
| Operator | Fire Departments* | 67 | 4 (1-7) | 0.83 (0.66-1.04) | 0.11 |
|  | Falck | 365 | 5 (3-8) | 1.00 (-) | - |
| Presence of paramedic | Yes | 190 | 4 (2-7) | 0.79 (0.68-0.93) | 0.0031 |
|  | No | 242 | 5 (2-9) | 1.00 (-) | - |
| Presence of relative | Yes | 159 | 5 (2-8) | 1.03 (0.88-1.20) | 0.75 |
|  | No | 273 | 4 (2-8) | 1.00 (-) | - |
| Vomit | Yes | 35 | 5 (3-9) | 1.10 (0.85-1.43) | 0.47 |
|  | No | 397 | 5 (2-8) | 1.00 (-) | - |
| Presence of trainee | Yes | 38 | 5 (3-8) | 0.85 (0.65-1.11) | 0.22 |
|  | No | 394 | 5 (3-8) | 1.00 (-) | - |

**Table S6 legend and footnotes**

The analyses were adjusted for all variables listed above as well as the timing of second IV access. The analysis of the first IV access was not adjusted for timing of the second IV access. Second IV access is not listed, as no model to isolate the effect of the second IV access could be developed.

The number of observations in each group includes only those included in the multivariate analysis (i.e. only those with complete data in all the variables used in the analysis). Thus, all 520 observations were not necessarily used.

*** Fire Departments:** Fire Department of Copenhagen and Fire Department of Frederiksberg
